# Bright-field HER2 dual in situ hybridization (DISH) assay on breast cancer cell blocks: a comparative study with histological sections

**DOI:** 10.1007/s12282-015-0664-1

**Published:** 2016-01-08

**Authors:** Rieko Nishimura, Nami Okamoto, Masakazu Satou, Kenta Kojima, Shinichi Tanaka, Natsumi Yamashita

**Affiliations:** 1 Department of Clinical Laboratory, National Hospital Organization Shikoku Cancer Center, 160 Kou, Minamiumemoto-machi, Matsuyama, Ehime 791-0280 Japan; 2 Division of Clinical Biostatistics, Section of Cancer Prevention and Epidemiology, Clinical Research Center, National Hospital Organization Shikoku Cancer Center, Matsuyama, Japan

**Keywords:** Breast cancer, Cytology, Cell block, *HER2* gene, DISH

## Abstract

**Background:**

HER2 testing for samples from recurrent or metastatic disease is recommended by the 2013 update of the American Society of Clinical Oncology/College of American Pathologists (ASCO/CAP) guidelines and cytological analysis can be applied to several types of metastatic lesions. However, the practical method to assess the HER2 testing of breast cancer cytology specimens has yet to be resolved. Therefore, we conducted the bright-field HER2 dual in situ hybridization (DISH) assay on cell blocks (CBs) prepared from breast cancer cell samples as a validation study before clinical use.

**Methods:**

CBs were prepared from tumor cell samples collected from 54 surgically excised breast tumors. The cells were fixed in 10 % buffered formalin for 16–28 h, and embedded in paraffin. The INFORM HER2/neu Dual ISH DNA Probe Cocktail was used for the DISH assay on the Ventana BenchMark ULTRA (Roche Diagnostics).

**Results:**

Successful results were obtained in 51 of 54 CB specimens, and the results from the CB specimens were in agreement with those from the histological sections in 48 of the 51 cases (concordance rate, 94 %; kappa, 0.846). The intraclass correlation coefficient (ICC) between the CB and histological specimens in the continuous HER2/CEP17 signal count ratio was 0.89 (95 % CI 0.81–0.93), and the Pearson’s CC was 0.91 (95 % CI 0.85–0.94).

**Conclusion:**

The HER2 DISH assay, utilizing 10 % buffered formalin-fixed CB, would be a reliable and ideal method to assess the HER2 gene status of breast cancer cytological specimens.

## Introduction

HER2 testing for samples from recurrent or metastatic disease is recommended by the 2013 update of the American Society of Clinical Oncology/College of American Pathologists (ASCO/CAP) guideline [1], due to the possibility that HER2 status may differ in recurrent disease. Cytological analysis can be applied to several types of metastatic lesions, as well as body cavity fluids, and is useful for patients who are in poor condition.

Several studies have reported good correlations between hormone receptor status in several types of cytological specimens, with their corresponding histological sections [2–4]. However, there are issues that remain to be resolved regarding HER2 testing for cytological specimens.

Immunocytochemical detection of HER2 protein overexpression in cytological specimens is unreliable due to unstable staining [5–11]. Although HER2 gene amplification visualization in cytological specimens by fluorescence in situ hybridization (FISH) demonstrates strong and consistent correlation with the HER2 status of the tissue samples [5, 6, 12, 13], there are some limitations to the FISH assay, such as the need of dark-field fluorescence microscopy and the lack of morphological details.

To overcome some of these limitations, the bright-field HER2 dual in situ hybridization (DISH) assay was developed. There are only a few reports of HER2 gene detection in cytological specimens using the bright-field HER2 DISH assay [14–18]. At the view of this, we need a preliminary validation study for the DISH assay to find a suitable method for cytological specimens before this method can be adopted in routine clinical practice.

Here, we conducted the DISH assay on cell blocks (CBs) prepared from cancer cell samples collected from surgically excised breast cancers, and compared the results with those from the corresponding histological sections.

## Materials and methods

CBs were prepared from tumor cell samples collected from 54 surgically excised breast tumors. Approximately 4-μm-thick sections were prepared on silanized glass slides from the CBs and the corresponding tissue blocks; the DISH assay and IHC staining were then performed on both the CB and tissue slides. The assay and staining were performed with a Ventana BenchMark ULTRA (Roche Diagnostics, Basel, Switzerland). The 2013 ASCO/CAP criteria for HER2 testing in breast cancer [1] was used to categorize the results. Two cases were excluded due to the small number of cells on the slide, and one case was excluded due to assay failure; therefore, 51 cases were included in the statistical analysis.

### Preparation of CBs

A single specimen was collected from each tumor using a 21-gauge needle attached to a 20-ml syringe mounted on an aspiration gun. The cells were fixed in 10 % buffered formalin for 16–28 h, and embedded in paraffin according to routine procedures.

### Preparation of histological specimens

Representative sections were prepared from the cut surface of the resected breast tumors. Tissues were fixed in 10 % buffered formalin for 24–48 h, and embedded in paraffin according to routine procedures.

### Histological breast cancer types

The following tumors were included: 49 invasive ductal carcinomas of no special type, two invasive lobular carcinomas, two noninvasive ductal carcinomas, and one mucinous carcinoma.

### DISH assay

The INFORM HER2/neu dual ISH DNA Probe Cocktail assay was performed on both the CB and tissue sections using the Ventana BenchMark ULTRA (Roche Diagnostics, Basel, Switzerland). The DISH assay was performed according to the manufacturer’s recommended protocol for surgical specimens. The standard protocol was initially performed for both types of sections; however, the protease reaction time was extended if the signals were weak. The HER2/neu (black) to chromosome enumeration probe 17 (CEP17) (red) ratio was manually counted using a light microscope in each specimen by one of the authors (NM), and the results confirmed by another author (RN). At least 20 cells were counted.

### Evaluation of the DISH results

The 2013 ASCO/CAP criteria for dual-color in situ hybridization (ISH) [1] were used to categorize both the CB and tissue section slides. The criteria consist of the combination of the HER2/CEP17 ratio and the average number of HER2 signals per cell. HER2 gene amplification was scored as “amplified” if the case had a HER2/CEP17 signal count ratio of 2.0, or, if the HER2/CEP17 signal count ratio was <2.0 but the average number of HER2 signals per cell was 6.0; “equivocal” if the case had a HER2/CEP17 signal count ratio of <2.0 and the average number of HER2 signals per cell was 4.0 and <6.0; and “not amplified” if the case had a HER2/CEP17 signal count ratio of <2.0 and the average number of HER2 signals was <4.0. CB results were compared with the tissue results from the same case.

### Data management

The Cohen’s kappa coefficient was used to assess the correlation between the results from the CBs and those from the tissue specimens both in the DISH assay and IHC staining. The correlation was scored as “good” if the kappa value exceeded 0.6 and “excellent” if it exceeded 0.8.

The intraclass correlation coefficient (ICC) and the Pearson’s correlation coefficient (CC) with a 95 % confidence interval (CI) were used on the continuous HER2/CEP17 signal count ratio to estimate the agreement between the CB and tissue specimens.

The Cohen’s kappa coefficient was calculated by Microsoft Office Excel 2013 software, and the ICC and CC were calculated using the irr package in the R statistical software: (http://cran.r-project.org/web/packages/irr/irr.pdf).

## Results

### Comparison of HER2 DISH results in CB and histological sections

**Fig. 1:**
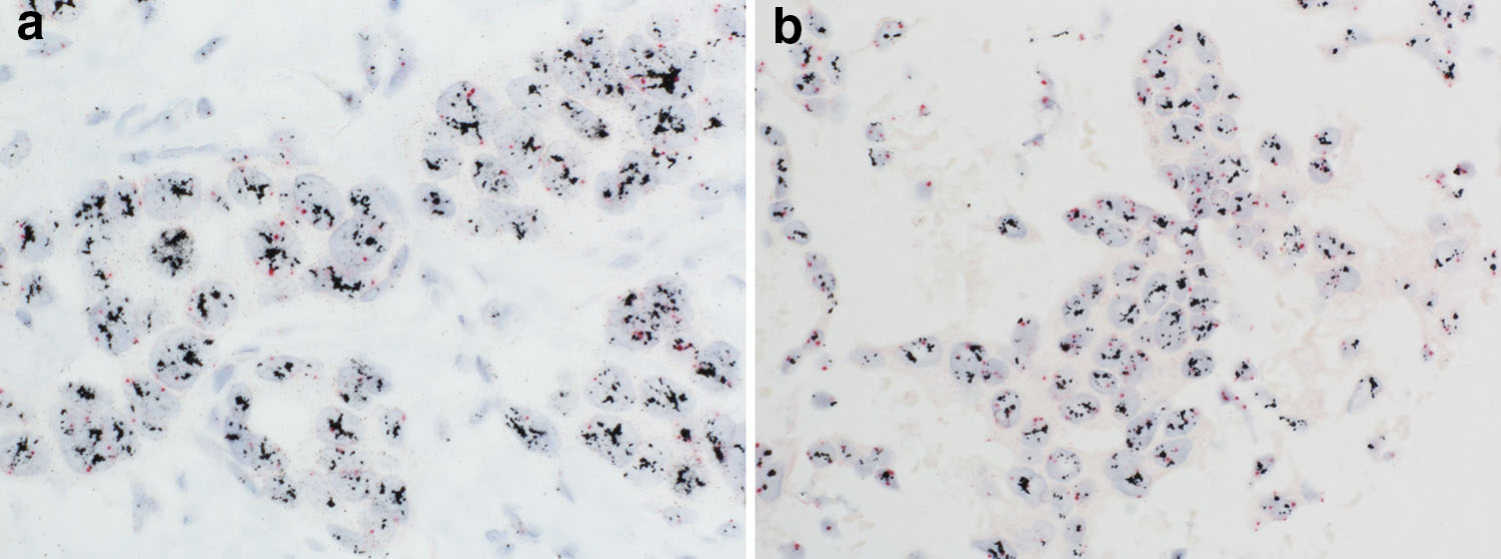
An example of a case with HER2 gene amplification showing consistent results between the histological specimen and cell block. The HER2/CEP17 ratio is 9.8 in the histological specimen (**a**) and 6.1 in the cell block (**b**)

**Fig. 2:**
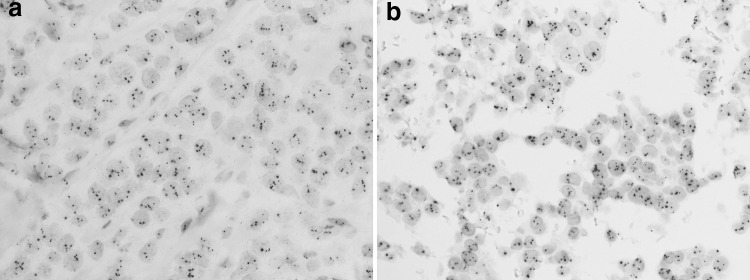
An example of a case with no amplification of the HER2 gene showing consistent results between the histological specimen and cell block. The HER2/CEP17 ratio is 1.2 in both the histological specimen (**a**) and the cell block (**b**)

As shown in Table 1, of the 51 cases analyzed, 48 cases showed agreement between the DISH results for the CB specimens and the corresponding histological sections (concordance rate, 94 %; kappa, 0.846). Ten cases showed HER2 amplification (Fig. 1), while 37 did not show HER2 amplification in either the CB or histological sections (Fig. 2). One case showed equivocal in both the CB and histological section. There were three discrepant cases, as follows: amplified in the CB but not amplified in the histological section, not amplified in the CB but amplified in the histological section, and equivocal in the CB and not amplified in the histological section.

**Table 1:** Comparison of HER2 DISH results from cell blocks and histological sections

Cell block	Histological section			
	Amplified	Equivocal	Not amplified	Total
Amplified	10	0	1	11
Equivocal	0	1	1	2
Not amplified	1	0	37	38
Total	11	1	39	51

*DISH* dual in situ hybridization

**Fig. 3:**
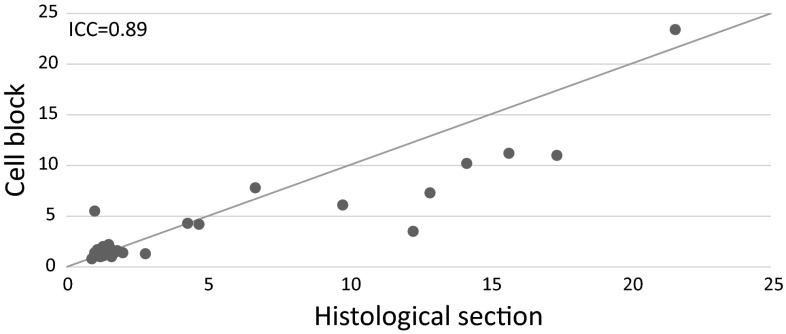
Distribution of the continuous HER2/CEP17 signal count ratio in the histological specimens and cell blocks. The two types of specimens showed good agreement when the ratio was below 2.0

The CBs and histological sections showed good agreement in the continuous HER2/CEP17 signal count ratio (Fig. 3); the ICC was 0.89 (95 % CI 0.81–0.93), and the Pearson’s CC was 0.91 (95 % CI 0.85–0.94).

## Discussion

Evaluation of the HER2 receptor status at metastatic sites is important for selecting the correct chemotherapy for treatment of recurrent disease [1]. Therefore, the use of cytology can be the only mode of tissue collection in the cases of metastatic lesion from which biopsies are difficult to obtain, a particular example being from body cavity fluids. Several studies have reported good correlations between hormone receptor status in several types of cytological specimens with their corresponding histological sections [2–4]. However, issues remain in HER2 testing using cytological specimens in routine clinical practice, both in the immunocytochemical detection of HER2 overexpression, as well as in gene amplification visualization by fluorescence in situ hybridization (FISH).

Discordant results have been reported using immunocytochemical detection of HER2 protein overexpression in several cytological specimen types, including CBs [5–11]. In a preliminary study at our institution, we encountered difficulty in scoring HER2 status by immunostaining using liquid-based cytological specimens stored in ThinPrep PreserveCyt Solution (Hologic), and found a low concordance rate with the histological sections (data not shown).

While several reports of FISH-based analyses using cytological specimens have demonstrated its accuracy and sensitivity [5, 6, 12, 13], this method has some disadvantages. In the FISH assay, morphological features are difficult to visualize under dark-field imaging. Therefore, the FISH assay is not suitable in clinical use for cytological specimens from body cavity fluids or aspirates, due to the presence of several non-neoplastic inflammatory cells. In addition, the FISH assay requires fluorescence imaging; however, fluorescence signals often fade quickly, unable to provide a permanent record.

There have been a few studies utilizing the HER2 DISH assay on liquid-based cytology specimens using the ThinPrep technique [14–16] and CBs [17, 18]. However, because the fixatives and fixation times were not well controlled in these previous studies, the DISH assay protocol required modification for adaptation to the particular cytological material being used.

There are two studies utilizing the HER2 DISH assay on CBs [17, 18]. Fritzsche et al. compared CB DISH results with those from CB FISH and histological specimens [17], although the authors did not compare CB specimens with histological specimens using DISH, and their samples included both primary and metastatic sites. Hartman et al. performed a pilot study of the DISH assay on CBs from 18 samples [18]. They compared three types of CBs using different fixatives, and the results were compared with the HER2 status determined by immunostaining or FISH; however, no comparison was performed between the CB DISH results to the histological specimen. Therefore, the present study is the first to compare the HER2 DISH assay on CBs from breast cancer primary sites with the corresponding histological sections.

The present study used cell samples fixed in 10 % buffered formalin for 16–28 h and embedded in paraffin. The fixation time was determined by preliminary testing, and to accommodate typical clinical working schedules. CBs were chosen because they are better suited for use in routine clinical practice, compared with cytological smears; formalin fixation and paraffin embedding are routinely used for processing histological sections in pathology laboratories. Because of the variation in fixation times in the previous studies using CBs, modification of the reported protocols was necessary to obtain slides with cells in good condition and sufficient signal; the same protocol was then used for both the CB and tissue sections in our study.

There was a strong correlation between the CB and histological HER2 DISH results; however, there were three discrepant cases. The discrepancy of all cases was attributable to differences in the distribution of the amplified cells between the CBs and histological specimens.

In summary, the DISH assay using CBs fixed in 10 % buffered formalin with an adequate fixation time would be the ideal method to assess the HER2 gene status of breast cancer cytology specimens.

This study was approved by the ethics committee at Shikoku Cancer Center on May 8 in 2014 (No. 19).
